# The Association between Secondhand Smoke and Stress, Depression, and Suicidal Ideation in Adolescents

**DOI:** 10.3390/healthcare9010039

**Published:** 2021-01-04

**Authors:** Eunmi Lee, Ka Young Kim

**Affiliations:** 1 Department of Nursing, Research Institute for Basic Science, Hoseo University, Asan 31499, Chungcheongnam-do, Korea; sweetbear2@hanmail.net; 2 Department of Nursing, College of Nursing, Gachon University, Incheon 21936, Korea

**Keywords:** secondhand smoke, SHS, stress, depression, suicidal ideation, adolescent

## Abstract

Background: Secondhand smoke (SHS) is an important risk factor for adolescents’ health. Several studies have reported that SHS is as dangerous as active smoking. Therefore, this study aimed to investigate the association between exposure to SHS and mental health, including stress, depression, and suicidal ideation, in adolescents. Methods: Using raw data from the 2018 14th Korea Youth Risk Behavior Web-Based Survey, we analyzed the effects of sociodemographic characteristics on stress, depression, suicidal ideation in 51,500 students, including 85.8% of all sampled students (n = 60,040), after excluding students with a history of smoking, and then we performed logistic regression analysis to determine the level of exposure to SHS and its impact on stress, depression, and suicidal ideation. Results: The increased level of exposure to SHS was positively associated with stress, depression, and suicidal ideation. Furthermore, stress, depression, and suicidal ideation increased as the level of SHS increased, after adjusting for variables such as age, gender, education level of the father and mother, school achievement, economic status, inhabitation, and drinking. Conclusions: This study demonstrates that SHS is positively associated with risk of mental health problems, including stress, depression, and suicidal ideation, in adolescents. Further research and policy strategies and systems to prevent and manage exposure to SHS in adolescents are required.

## 1. Introduction

Secondhand smoke (SHS) is defined as unwanted smoke that people breathe in from cigarettes other people are smoking [1]. Cigarette smoking is well known as a major cause of death from respiratory diseases, including lung cancer and chronic obstructive pulmonary disease, cardiovascular diseases such as coronary heart disease, and various cancers including bladder, blood, cervix, colon, and kidney cancer [2]. However, several studies have reported that SHS is as dangerous as active smoking because nonsmokers who inhale SHS are affected by the same harmful chemicals as active smokers [1,2,3].

SHS is reported to be associated with mental health problem in various epidemiologic studies [4,5,6,7]. Furthermore, cigarette components are known to affect mental health. Like active smoking, SHS consists of several noxious chemicals, including nicotine, carbon dioxide, carbon monoxide, carbonyls, hydrocarbons, polycyclic aromatic hydrocarbons, nitrosamines, and tar [8]. Among them, nicotine is the main psychoactive constituent of tobacco. It has been reported that nicotine is affected by activating or acting as an agonist for the acetylcholine, nicotinic acetylcholine receptors [9,10]. Several preclinical and clinical studies have demonstrated that nicotine affects mood, anxiety, aggression, and related behaviors, such as irritability and agitation [9]. The role of nicotinic acetylcholine receptors in regulating mood and anxiety has been elucidated based on the effects of nicotine on aggression-related behavioral states in animal models. Thus, smoking is closely associated with mental health problems and neuropsychiatric disorders [11,12].

SHS is an important factor for children and adolescents’ health. Children and adolescents are generally exposed to SHS via indoor air at home, in school, and in public places, such as stores, restaurants, internet cafes, concert halls, or on the street. Some teenagers are beginning to smoke in their early years of life [13]. According to a Centers for Disease Control and Prevention report, approximately 40.6% of US children are exposed to SHS [2]. SHS has been known to cause various health problems in children, including low birth weight, asthma, respiratory infections, ear infections, and sudden infant death syndrome [8,14]. Adolescents that transit from childhood to adulthood undergo physical, various social, and psychological changes and are exposed various risk factors such as alcohol, smoking, and substance use [15,16]. In particular, exposure to toxic chemical such as smoking makes adolescents vulnerable to health problems.

Adolescence is an important period for mental health [17,18]. Multiple factors including genetic, biological, environmental, and cultural considerations are associated with risk of mental health in adolescence [19,20]. Stress is a critical risk factor for substance abuse and can lead to anxiety and depression [21,22]. Exposure to SHS is reported to be a risk factor for high stress in both smoking and non-smoking adults [1]. Globally, depression is one of the most common mental health problems in adolescents. SHS exposure is associated with depressive symptoms in young people [23]. Furthermore, suicide is the third cause of death in older adolescents (15~19 years) [2]. The risk factors for suicide are associated with alcohol and drug abuse, a suicidal personal history or family history, mental illness, negative self-conception and isolation, and self-destructive behaviors [24]. However, the effects of SHS exposure on the mental health of adolescents are still unclear. Moreover, it is critical issue, and continuous research on the association between SHS exposure and mental health in adolescents is required.

Therefore, in this study, we hypothesized that adolescents’ exposure to SHS could be associated with their mental health, including stress, depression, and suicidal ideation. This study aimed to investigate the association between exposure to SHS and mental health, including stress, depression, and suicidal ideation, in adolescents.

## 2. Materials and Methods

### 2.1. Design

This descriptive cross-sectional study used secondary data from the 2018 14th Korea Youth Risk Behavior Web-Based Survey (KYRBWS-XIV) to determine the association between the level of exposure to secondhand smoking on stress, depression, and suicidal ideation among Korean adolescents.

### 2.2. Study Population

We used open data from KYRBWS-XIV provided by the Korea Centers for Disease for analysis. KYRBWS-XIV is an anonymous self-reported online survey conducted from 1–30 June of 2018 with middle and high school students (grades 7–12) across the country. In a two-stage cluster sampling design, schools were selected as primary sampling units (PSUs) by stratifying students by school type and district, and classes were selected as secondary sampling units (SSUs). All students of the sampled classes (SSUs) were included in the survey, except for those with absenteeism, special needs, or reading disability. As a result, a total of 62,823 students in 800 schools (400 high schools and 400 middle schools) were selected for the survey, of whom, 60,040 participated in the survey, making the participation rate 95.6% (Ministry of Education, 2018). After excluding students with a history of smoking, given the possible impact of past smoking on the effect of secondhand smoking, 51,500 students (85.8%) were included in the study.

### 2.3. Variables

The level of exposure to SHS was measured based on the total score of the answers to the following questions: Over the last 7 days, how often (number of days) were you near smokers at home (family members or guests)?; did you smell tobacco smoke from someone else’s cigarette in an indoor environment at school (classroom, restroom, hallway, etc.)?; did you smell tobacco smoke from someone else smoking in an indoor environment other than home and school (store, restaurant, shopping mall, concert hall, internet cafe, etc.)? The answers were rated by the number of days from 0 (never) to 7 (everyday). The score (number of days) was defined as the level of exposure to SHS, and the total score was used for the analysis. Although the questions required an answer to “how many days per week,” this does not mean the entire day but rather the number of days on which the respondent was exposed during that day, regardless of frequency, and no intersection could occur because each question has a different place of exposure. In order for the number of days and the place of exposure to be considered simultaneously, the level of exposure was calculated based on the sum of all three variables, which ranged from 0 (never in all three occasions) to 21 (every day in all three occasions).

Stress was measured based on the answers to the question “How much stress do you usually feel?” as quantitative variables rated on a 5-point Likert scale from 1 (not at all) to 5 (extremely). The answers were analyzed as binary variables by converting “not at all” (1) and “very slightly” (2) to 0 and “moderately” (3), “strongly” (4), and “extremely” (5) to 1.

Depression was measured by the answers to the question “Over the last 12 months, have you ever felt sad or despaired to the point of withdrawing from daily activities for two consecutive weeks?” as binary variables (0 = No, 1 = Yes), which were used for the analysis without further processing.

Suicidal ideation was measured by the answers to the question “Over the last 12 months, have you had thoughts about suicide?” as binary variables (0 = No, 1 = Yes), which were used for the analysis without further processing.

We used the respondents’ general characteristics (age, gender, education level of parents, school achievement, economic status, inhabitation, and drinking) for the analysis model as control variables, based on the results of the data analysis of previous studies as well as our study, that can influence the study results. For education level of parents, education level of father and education level of mother were used as binary variables for high school graduation or lower and university enrollment or higher, respectively. School achievement was used as a quantitative variable, which is a Likert 5-point scale, with the question of “What is your academic performance in the last 12 months?” and economic status with the question “How is your family’s economic status?”. Inhabitation was assessed with the question “what is your current residence type?” and options “I live with my family”, “I live in a relative’s house”, “Lodging, self-catering (including living with friends)”,”Dormitory”, “Childcare”. Five measures were measured, including facilities (orphanage, social welfare facility, nursery school). In this study, inhabitation is converted to “with family” and “without family” for responding to “living with family” and “living in a relative’s house”, and is considered as a binary variable and used for analysis. In the case of drinking, the values measured as “no” and “yes” were used for the question “Have you ever drunk more than one drink?”.

### 2.4. Statistical Analysis

Since KYRBWS-XIV was a survey based on a complex sampling design using two-stage stratified cluster sampling, the analysis was performed as complex sample estimates using weight (proc surveyfreq, proc surveylogistic, etc. using SAS 9.4(SAS Institute Inc., Cary, NC, USA)). Logistic regression analysis was performed to analyze the association between the level of exposure to SHS and stress, depression, and suicidal ideation using the respondents’ general characteristics of age, gender, education level of parents, school achievement, economic status, inhabitation, and drinking as the control variables; the level of exposure to SHS was used as the independent variable; and stress, depression, and suicidal ideation as the dependent variables.

## 3. Results

### 3.1. General Characteristics and Their Effects on Stress, Depression, and Ideation

Of the 51,500 smokers, 81.6% were feeling a little stress in their normal lives, 25.5% had felt enough sadness or despair to stop their daily activities for two consecutive weeks in the last year, and 12.4% were seriously depressed in the last year. There was a time when I wanted to commit suicide. As a result of investigating the variations in the prevalence of stress, depression, and suicidal ideation according to each of the general characteristics, it was found that most of the variables had statistically significant effects on stress, depression, and suicidal ideation (Table 1). Older age was associated with a higher prevalence of stress and depression, but its effect on the prevalence of suicidal ideation was not statistically significant. As for gender difference, female students had a higher prevalence of stress, depression, and suicidal ideation than male students. In terms of the education level of parents, stress was found to increase with higher education levels of both parents, but its effect on the prevalence of depression and suicidal ideation was not statistically significant. Higher school achievement and economic status were found to be associated with a lower prevalence of all three dependent variables (stress, depression, and suicidal ideation). In terms of the variable of inhabitation, students “without family” showed a higher prevalence of stress and depression when compared to those “with family,” but no statistically significance intergroup difference was observed for suicidal ideation. As for drinking, students with experience showed a higher prevalence of stress, depression, and suicidal ideation than those without.

### 3.2. Association between Secondhand Smoke and Stress, Depression, and Suicidal Ideation

Table 2 presents the results of a logistic regression analysis conducted to analyze the association between the level of exposure to SHS and the prevalence of stress, depression, and suicidal ideation. Model 1 is a simple logistic regression model employed to analyze the association between the exposure to SHS and the prevalence of stress, depression, and suicidal ideation. Model 2 is the result of multiple logistic regression analysis using the respondents’ general characteristics (age, gender, education level of parents, school achievement, economic status, inhabitation, and drinking) as the control variables.

The level of exposure to SHS was positively related to stress, that is, the higher the level of exposure to SHS, the higher the prevalence of stress (odds of stress: 1.09-fold). Application of general characteristics as control variables slightly mitigated the effect of the level of exposure to SHS on stress (odds of stress: 1.07-fold). The level of exposure to SHS was positively related to depression, that is, the higher the level of exposure to SHS, the higher the prevalence of depression (odds of depression: 1.10-fold). Application of general characteristics as control variables slightly mitigated the effect of the level of exposure to SHS on stress (odds of depression: 1.08-fold). The level of exposure to SHS was positively related to suicidal ideation, that is, the higher the level of exposure to SHS, the higher the prevalence of suicidal ideation (odds of suicidal ideation: 1.09-fold). Application of general characteristics as control variables slightly mitigated the effect of the level of exposure to SHS on suicidal ideation (odds of stress: 1.07-fold).

## 4. Discussion

This study demonstrated the association between SHS exposure and risk of mental health problem including stress, depression, and suicidal ideation in adolescents. Furthermore, exposure to SHS is positively associated with risk of stress, depression, and suicidal ideation in adolescents after adjusting for significant variables.

Reportedly, exposure to SHS has been demonstrated as a risk factor for high stress in both smokers and non-smokers at work and in the home [1]. The non-smoker group exposed to SHS had a higher pooled annual prevalence of having a major depressive episode when compared to non-smokers without SHS in Canada [3]. SHS is positively associated with mental health issues, such as major depressive disorder, generalized anxiety disorder, attention-deficit/hyperactivity disorder, and conduct disorder, particularly in children and adolescents [4]. Adolescence is a period of significant changes and of rapid physical, psychological, and social development, and adolescents are highly exposed and vulnerable to environmental stresses, such as frequent conflicts with family and friends, academic performance, and changes in physical appearance as well as rapid neuroendocrine changes. These physical, emotional, and social changes make adolescents vulnerable to mental health problems.

Exposure to SHS affects several physical and mental health problems in adolescents; furthermore, it can affect them persistently until their adult lives. Some reports have demonstrated that exposure to SHS decreased lung function during childhood, leading to a reduced maximum level in adolescence [25,26]. SHS exposure during childhood is detrimental to arterial function and structure, resulting in premature atherosclerosis, impaired cardiac autonomic function, and changes in heart rate variability [8]. However, some reports have demonstrated that short-term exposure to SHS may not result in significant damage in humans; however, long-term exposure increased inflammation [27,28]. Patients persistently exposed to SHS had a higher risk of recurrent cardiac events after hospitalization when compared to patients who were not exposed to SHS [29]. SHS exposure may not lead to immediate damage and diseases in childhood and adolescents. However, persistent and chronic exposure to SHS can eventually cause various damage and diseases, including stroke and pulmonary and cardiovascular diseases, through direct or indirect pathogenesis in adults [8,26,30]. Several toxic chemicals from direct SHS exposure cause airway epithelium tissue destruction with acting carcinogens and oxidants and can indirectly cause damage through signaling pathways related to tissue cell repair and inflammation [27]. However, generally, mental health problems, including depression, anxiety, and suicidal ideation, must be affected immediately during the adolescence stage. Moreover, it can affect adolescents persistently until their adult lives. Early exposure to SHS during adolescence may eventually affect mental and physical health in adults through persistent and chronic exposure. Therefore, it is very important and urgent to prevent and manage short- and long-term exposure to SHS in adolescents.

The positive association between SHS exposure and mental health may be explained as integrating biological and epidemiological aspects [7,17,18,31]. The positive association between SHS exposure and mental health may be explained as integrating biological and epidemiological aspects [1,21,22,31,32,33]. Some studies have reported that exposure to SHS in stressful states for a long time is related to the risk for negative lifestyle and behavioral problems such as emotional and conduct disorders at a young age [21,22,31]. A negative lifestyle, manifest in lifestyle aspects such as physical exercise, eating habits, and sleep patterns, can exacerbate mental health problem [31]. Furthermore, nicotine reportedly causes physical changes such as increased heart rate, blood pressure, and a breathing rate similar to stress response [1,34]. Nicotine is associated with stress, mood, anxiety, depression, and aggression in preclinical and clinical studies [1,9,10]. SHS exposure reportedly increases levels of corticotrophin-releasing hormone and adrenocorticotropic hormone that is related to mood and behavior [31,35]. Thus, SHS is critical issue in adolescents vulnerable to mental health problems.

In the two models in this study, SHS was also a significant risk factor after adjusting for variables such as age, gender, education level of the father and mother, school achievement, economic status, inhabitation, and drinking, which have been known to affect adolescents. These variables are well known to affect depression, anxiety, and suicidal ideation in adolescents. Adolescents who have depression are reported to be positively associated with anxiety, conduct disorders, substance abuse, and suicidal tendency [36]. Moreover, this study has demonstrated that the level of exposure to SHS is associated with risk of mental health problems, including stress, depression, and suicidal ideation, in adolescents. The level of exposure to SHS is a significant risk factor for mental health problems during adolescence. However, it is impossible to pay attention and manage this only at the individual level. Strict management at home and school is required; furthermore, massive social and national policies are needed to prevent exposure to SHS for children and adolescents.

There are some limitations to this study. First, it is not possible to demonstrate the causality among the variables due to the cross-sectional study that analyzed the secondary data. Additional longitudinal studies need to be conducted. Second, the variables were confined to questionnaires based on the Korea Youth Risk Behavior Web-based survey, and variables with a single question were limited in detailed analysis and evaluation. To improve the validity of the research results, various additional studies, such as qualitative research on the extent and location of SHS exposure, major providers, and pathways related to exposure are required. Finally, it is difficult to establish a cause and effect relationship because the exposure and outcome are simultaneously assessed and because other time frames were used in this survey.

## 5. Conclusions

This study has demonstrated that SHS is positively associated with risk of mental health problems, including stress, depression, and suicidal ideation, in adolescents. Further research and policy strategies and systems to prevent and manage exposure to SHS in adolescents are required.

## Figures and Tables

**Table 1:** General characteristics and single effects on stress, depression, or suicidal ideation.

Characteristics		Total	Stress		Depression		Suicidal Ideation	
		% Mean ± SD	OR	Wald χ^2^ (*p*)	OR	Wald χ^2^ (*p*)	OR	Wald χ^2^ (*p*)
Age		15.0 ± 1.8	1.13	196.560 (<0.001)	1.07	63.840 (<0.001)	0.99	0.846 (0.356)
Gender	Male	48.2	74.9	2412.497 (<0.001)	18.6	1894.387 (<0.001)	8.2	762.843 (<0.001)
	Female	51.8	87.9		32.0		16.4	
Education of father	≤High school	69.8	81.5	9.815 (0.002)	26.0	0.039 (0.843)	12.5	0.442 (0.506)
	≥College	30.2	83.0		26.1		12.2	
Education of mother	≤High school	64.3	81.3	21.933 (<0.001)	26.3	0.412 (0.521)	12.5	0.447 (0.504)
	≥College	35.7	83.4		26.6		12.7	
School achievement (1–5)		3.2 ± 1.2	0.87	188.788 (<0.001)	0.85	295.496 (<0.001)	0.85	167.185 (<0.001)
Economic status (1–5)		3.4 ± 0.9	0.87	473.063 (<0.001)	0.84	215.796 (<0.001)	0.78	237.776 (<0.001)
Inhabitation	With family	96.1	81.5	10.128 (0.002)	25.4	7.307 (0.007)	12.4	2.415 (0.120)
	Without family	3.9	84.5		28.6		13.7	
Drinking	No	65.1	79.9	170.235 (<0.001)	22.0	592.980 (<0.001)	10.6	230.755 (<0.001)
	Yes	34.9	84.9		32.2		15.8	

**Table 2:** Association between level of exposure to secondhand smoke and stress, depression, and suicidal ideation.

Independent Variables	Stress		Depression		Suicidal Ideation	
	Model 1	Model 2	Model 1	Model 2	Model 1	Model 2
	OR (*p*)	OR (*p*)	OR (*p*)	OR (*p*)	OR (*p*)	OR (*p*)
Level of exposure to SHS	1.09 (<0.001)	1.07 (<0.001)	1.10 (<0.001)	1.08 (<0.001)	1.09 (<0.001)	1.07 (<0.001)
Age		0.92 (<0.001)		0.97 (0.670)		0.92 (<0.001)
Gender (ref = male)		2.09 (<0.001)		1.97 (<0.001)		2.09 (<0.001)
Education level of father (ref = college or higher)		0.84 (<0.001)		0.89 (<0.001)		0.84 (<0.001)
Education level of mother (ref = college or higher)		0.90 (0.007)		0.87 (<0.001)		0.90 (0.007)
School achievement (1–5)		0.87 (<0.001)		0.88 (<0.001)		0.89 (<0.001)
Economic status (1–5)		0.83 (<0.001)		0.91 (<0.001)		0.83 (<0.001)
Inhabitation (ref = with family)		1.24 (0.008)		1.20 (0.001)		1.24 (0.008)
Drinking (ref = no)		1.60 (<0.001)		1.30 (<0.001)		1.60 (<0.001)
Wald (*p*)	318.71 (<0.001)	144.85 (<0.001)	1058.47 (<0.001)	207.32 (<0.001)	648.27 (<0.001)	144.85 (<0.001)
Cox and Snell’s R-square	0.010	0.035	0.023	0.057	0.013	0.035
Nagelkerke’s R-square	0.017	0.066	0.033	0.083	0.025	0.066

Model 1: Simple logistic regression. Model 2: Multiple logistic regression with control variables such as age, gender, education level of parents, school achievement, economic status, inhabitation, and drinking.

## Data Availability

Not applicable.
